# Delayed Constrictive Pericarditis Following Presumed Esophagopericardial Communication After Esophageal Stenting

**DOI:** 10.7759/cureus.115555

**Published:** 2026-08-31

**Authors:** Shahana Alasgarli, Matin Hadizada, Ibrahim Rufullayev, Shovkat Javadova, Gulay Mammadova

**Affiliations:** 1 Cardiology, Yeni Klinika, Baku, AZE; 2 Cardiac Surgery, Yeni Klinika, Baku, AZE; 3 Internal Medicine, Yeni Klinika, Baku, AZE

**Keywords:** anterior pericardiectomy, bacterial pericarditis, chronic constrictive pericarditis, effusive-constrictive pericarditis, esophageal cancer (ec), esophago-pericardial fistula

## Abstract

Esophagopericardial fistula is a rare but life-threatening complication of esophageal stenting and malignancy. Although acute purulent pericarditis is a well-recognized consequence, delayed presentation as constrictive pericarditis years after the initial insult is exceptionally uncommon.

A 74-year-old woman presented with a two-month history of progressive dyspnea. She had undergone esophageal stent implantation for esophageal cancer two years earlier, followed by chemoradiotherapy. Transthoracic echocardiography demonstrated preserved biventricular systolic function and constrictive physiology without significant pericardial effusion. Chest computed tomography revealed dense pericardial fluid containing air bubbles, raising suspicion of an esophagopericardial communication. However, contrast-enhanced computed tomography with both oral and intravenous contrast failed to demonstrate an active fistulous tract. Cardiac magnetic resonance imaging confirmed constrictive physiology with septal bounce. The patient underwent surgical pericardiectomy, during which a large amount of firm, white, cheese-like purulent material was evacuated. Microbiological culture of the pericardial specimen grew *Enterobacter cloacae*, susceptible to piperacillin/tazobactam and meropenem. Histopathological examination demonstrated chronic inflammation, extensive fibrosis, and dystrophic calcification, confirming chronic constrictive pericarditis. Following surgical intervention and targeted antimicrobial therapy, inflammatory markers normalized, and the patient's heart failure symptoms resolved.

Delayed constrictive pericarditis secondary to a presumed spontaneously sealed esophagopericardial fistula represents an extremely rare late complication of esophageal stenting. The presence of intrapericardial air should prompt suspicion for prior esophagopericardial communication even when contrast imaging fails to demonstrate an active fistula. Early multimodality imaging, surgical pericardiectomy, and targeted antimicrobial therapy are essential for successful management.

## Introduction

Constrictive pericarditis is a chronic inflammatory disorder characterized by pericardial fibrosis and loss of pericardial compliance, resulting in impaired ventricular filling and symptoms of predominantly right-sided heart failure [1]. The condition may develop following cardiac surgery, radiation therapy, tuberculosis, viral infections, or less commonly, bacterial pericarditis. Because its clinical presentation often overlaps with restrictive cardiomyopathy, establishing the correct diagnosis requires careful integration of clinical findings and multimodality imaging [2,3].

Esophagopericardial fistula is an exceptionally rare but life-threatening condition that may occur secondary to esophageal malignancy, esophageal surgery, stent placement, or other esophageal interventions [4]. Most reported cases present acutely with purulent pericarditis, cardiac tamponade, or sepsis, requiring urgent surgical management. Delayed presentation with chronic constrictive physiology is exceedingly uncommon [4,5].

We report a rare case of constrictive physiology developing years after esophageal stent implantation. Although no active communication between the esophagus and the pericardial cavity was identified on contrast-enhanced computed tomography (CT), multimodality imaging demonstrated features of constrictive physiology together with pneumopericardium, suggesting a previously formed esophagopericardial fistula that had spontaneously sealed. This case highlights the importance of considering remote esophageal interventions in the differential diagnosis of unexplained constrictive physiology and emphasizes the diagnostic value of multimodality imaging in distinguishing constrictive physiology from restrictive cardiomyopathy [6].

## Case presentation

A 74-year-old woman presented to the emergency department with progressively worsening dyspnea over the preceding two months. On admission, her blood pressure was 107/70 mmHg, heart rate was 105 beats per minute, and oxygen saturation was 92% on room air. She was afebrile. Initial evaluation included chest radiography and electrocardiography. The electrocardiogram showed sinus tachycardia with no significant abnormalities. Chest radiography revealed bilateral pleural effusions (Figure 1).

**Figure 1:**
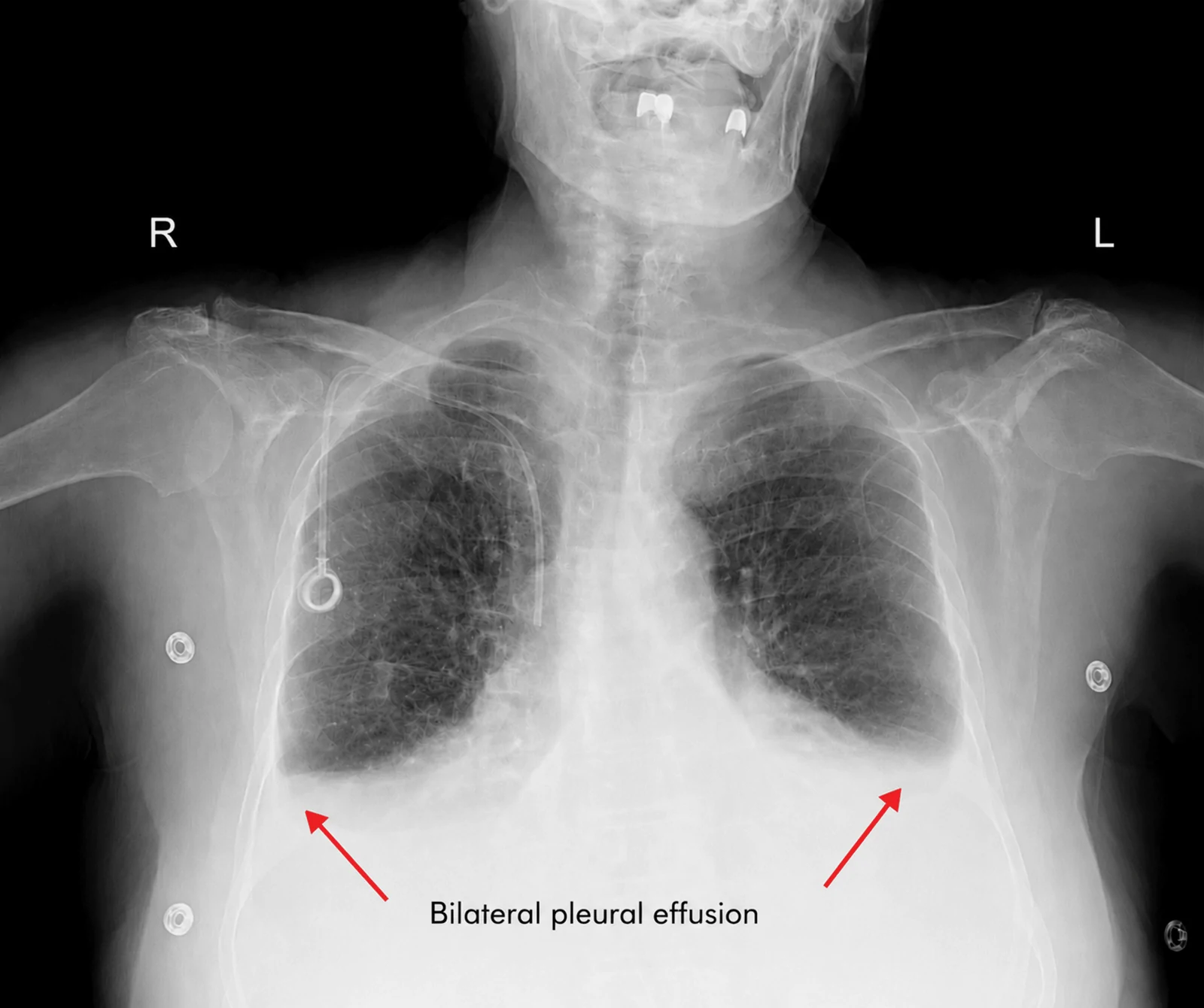
Anteroposterior chest radiograph showing bilateral pleural effusions (red arrows), more prominent on the left.

Subsequent detailed history-taking revealed that the patient had undergone esophageal stent implantation two years earlier due to esophageal cancer, followed by radiotherapy and chemotherapy. According to the patient, a pericardial effusion was noted at the time chemoradiotherapy was initiated two years ago, which was managed medically. However, the patient currently has no available medical records documenting this history, mode of radiotherapy, or chemotherapy.

Transthoracic echocardiography demonstrated preserved left and right ventricular systolic function and pericardial effusion with constrictive physiology, with no significant valvular abnormalities (Video 1, Figures 2-4 ).

**Video 1:** Transthoracic echocardiography demonstrating a dense pericardial effusion and septal bounce, consistent with constrictive physiology.

**Figure 2:**
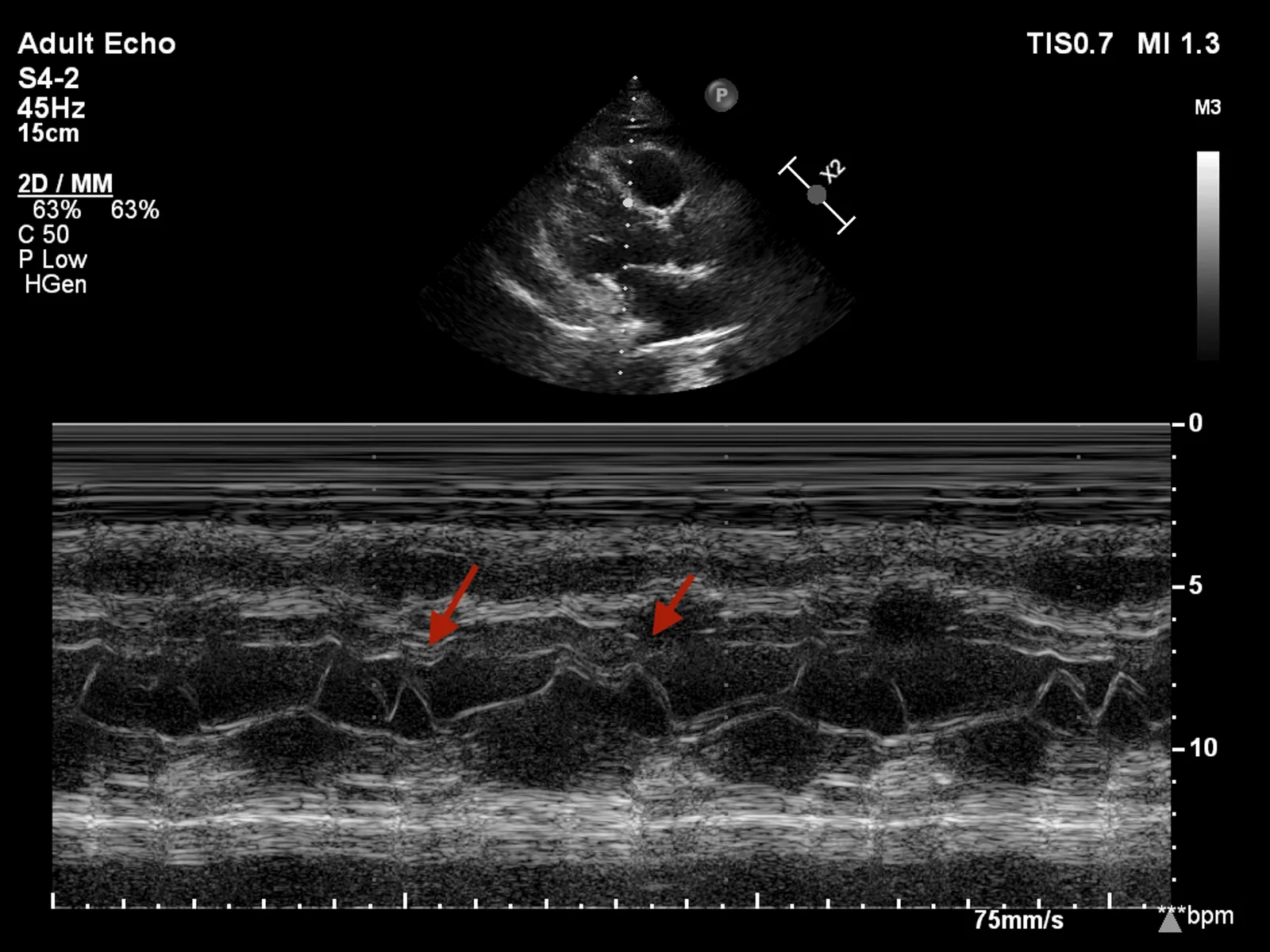
M-mode transthoracic echocardiography demonstrating septal bounce (abnormal early diastolic interventricular septal motion) (red arrows), consistent with constrictive physiology.

**Figure 3:**
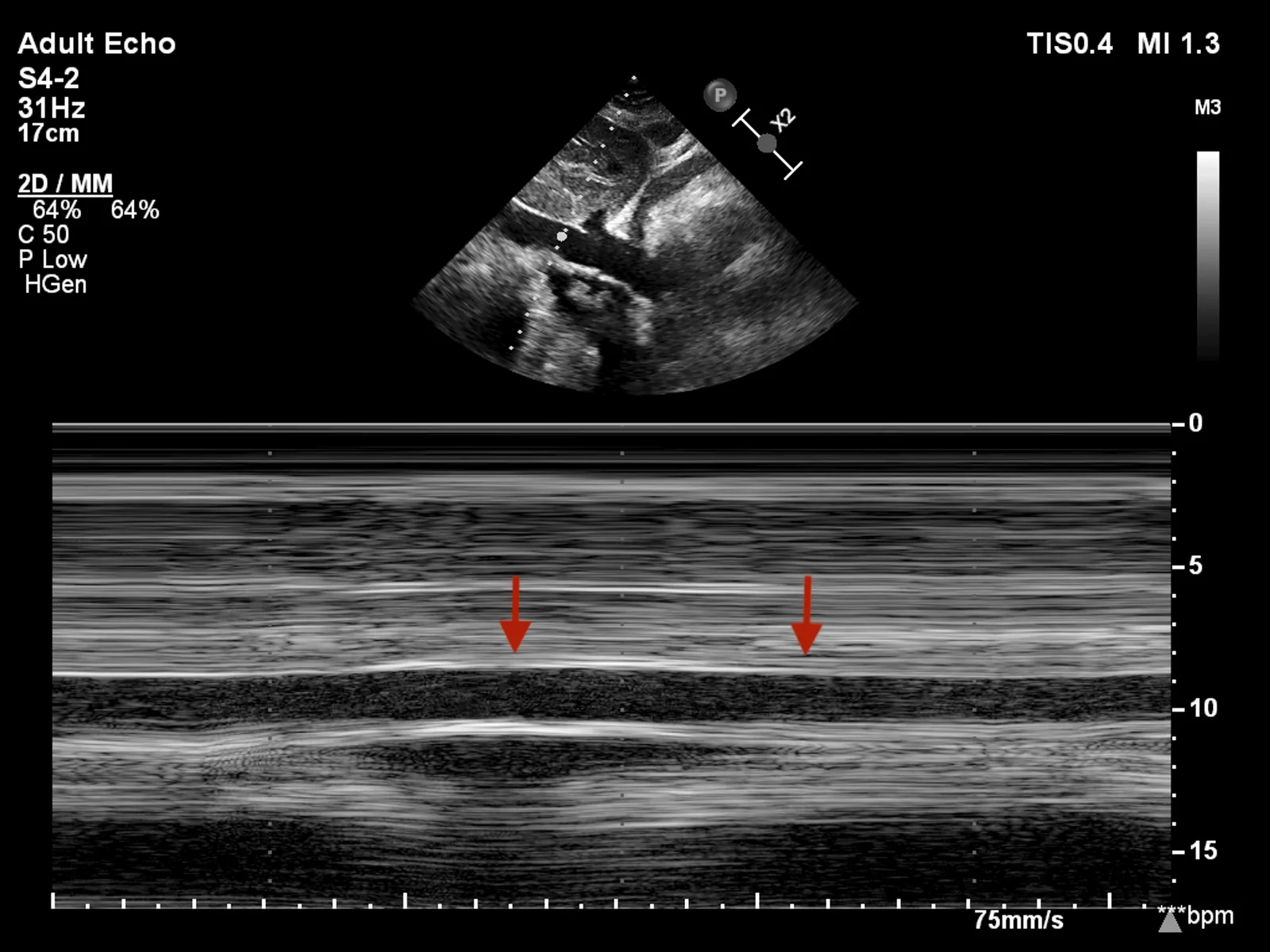
Subcostal M-mode transthoracic echocardiographic view demonstrating a dilated inferior vena cava with markedly reduced inspiratory collapse (arrows), consistent with elevated right atrial pressure.

**Figure 4:**
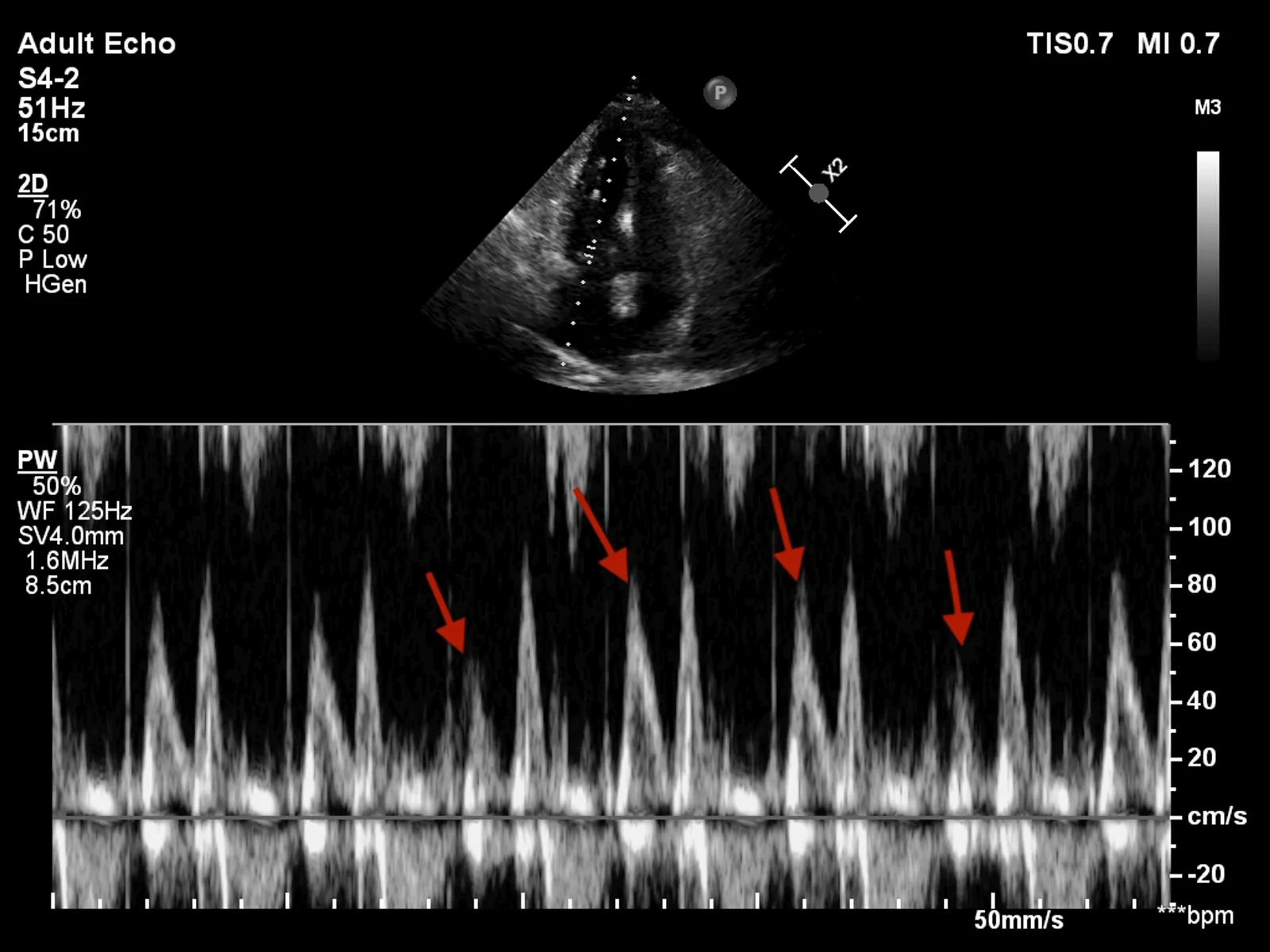
Pulsed-wave Doppler of the transmitral inflow demonstrating marked respiratory variation in early diastolic (E-wave) velocity (arrows), consistent with constrictive physiology.

Routine laboratory tests were performed upon admission. Apart from an elevated C-reactive protein (CRP), N-terminal pro-B-type natriuretic peptide (NT-pro BNP) levels, and leukocytosis, no significant laboratory abnormalities were detected. The laboratory test results are presented in Table 1.

**Table 1:** Laboratory test results on admission

Laboratory test (full name)	Result	Reference value
Hb (hemoglobin)	11.4	12.5-15.1 g/dL
Hct (hematocrit)	38	36%-47%
PLT (platelet count)	189	184-433 × 10^9^/L
WBC (white blood cell count)	22.33	5.2-9.2 × 10^9^/L
Creatinine (serum creatinine)	1.1	0.5-0.9 mg/dL
Potassium (K+, serum potassium)	3.9	3.5-5.1 mmol/L
Sodium (Na+, serum sodium)	137	135-145 mmol/L
Urea (serum urea)	53	15-40 mg/dL
BUN (blood urea nitrogen)	25	8-20 mg/dL
NT-pro BNP (N-terminal pro-B-type natriuretic peptide)	6136	<125 pg/mL
TSH (thyroid-stimulating hormone)	4.16	0.27-4.2 mIU/L
CRP (C-reactive protein)	98	<5 mg/L
AST (aspartate aminotransferase)	83	10-35 U/L
ALT (alanine aminotransferase)	45	7-35 U/L
CK-MB (creatine kinase-myocardial band)	41	<25 U/L
Albumin (serum albumin)	3.8	3.5-5.2 g/dL

To further clarify the diagnosis, a non-contrast chest CT scan was performed. CT imaging revealed dense pericardial fluid containing air bubbles within the pericardial cavity (Figure 5).

**Figure 5:**
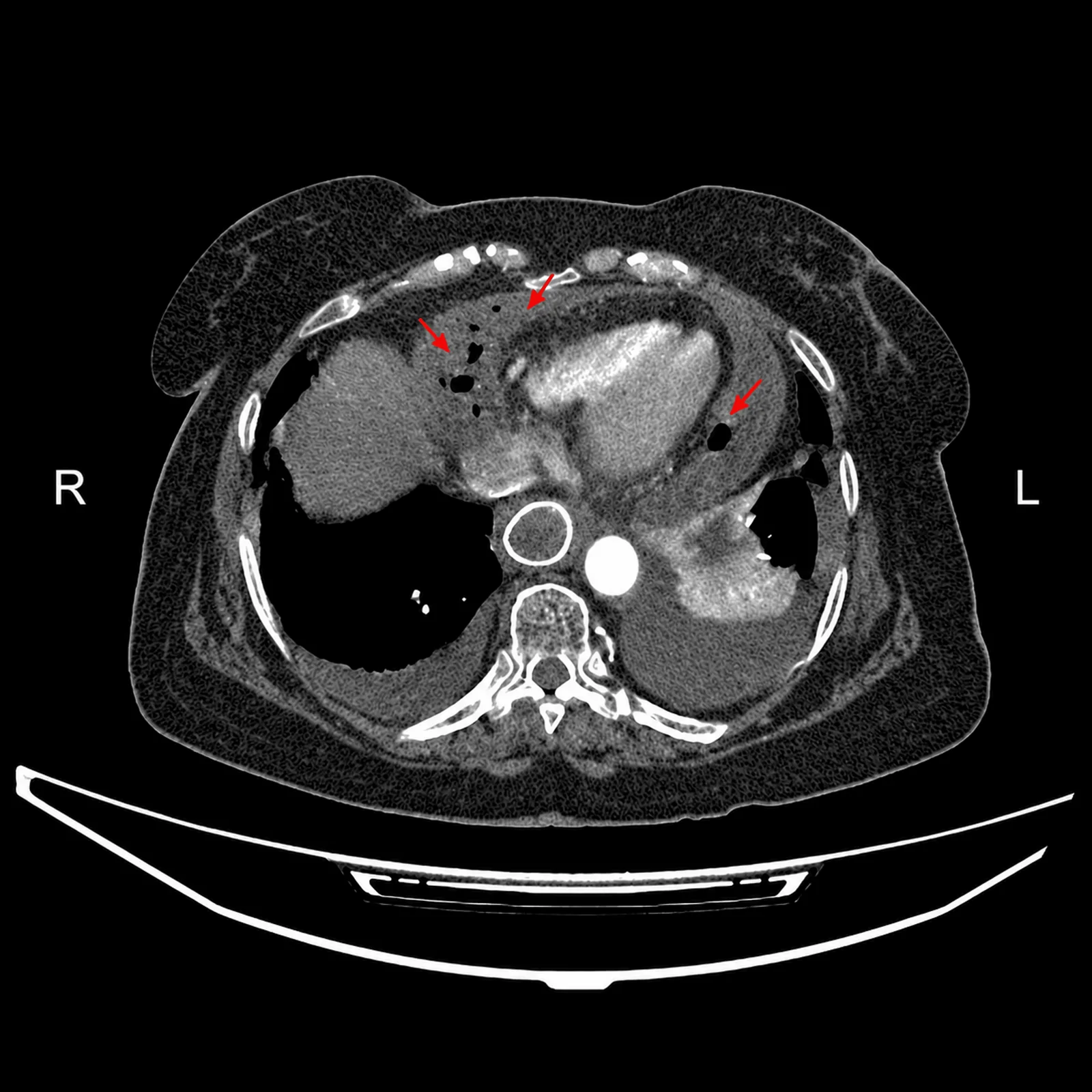
Axial contrast-enhanced computed tomography of the chest demonstrating multiple foci of air within the pericardial space (arrows), consistent with pneumopericardium, with associated bilateral pleural effusions.

Following the identification of air within the pericardial space, an esophagopericardial communication was strongly suspected. Therefore, a contrast-enhanced chest CT using oral contrast was performed; however, no evidence of communication between the esophagus and the pericardial cavity was identified (Figure 6).

**Figure 6:**
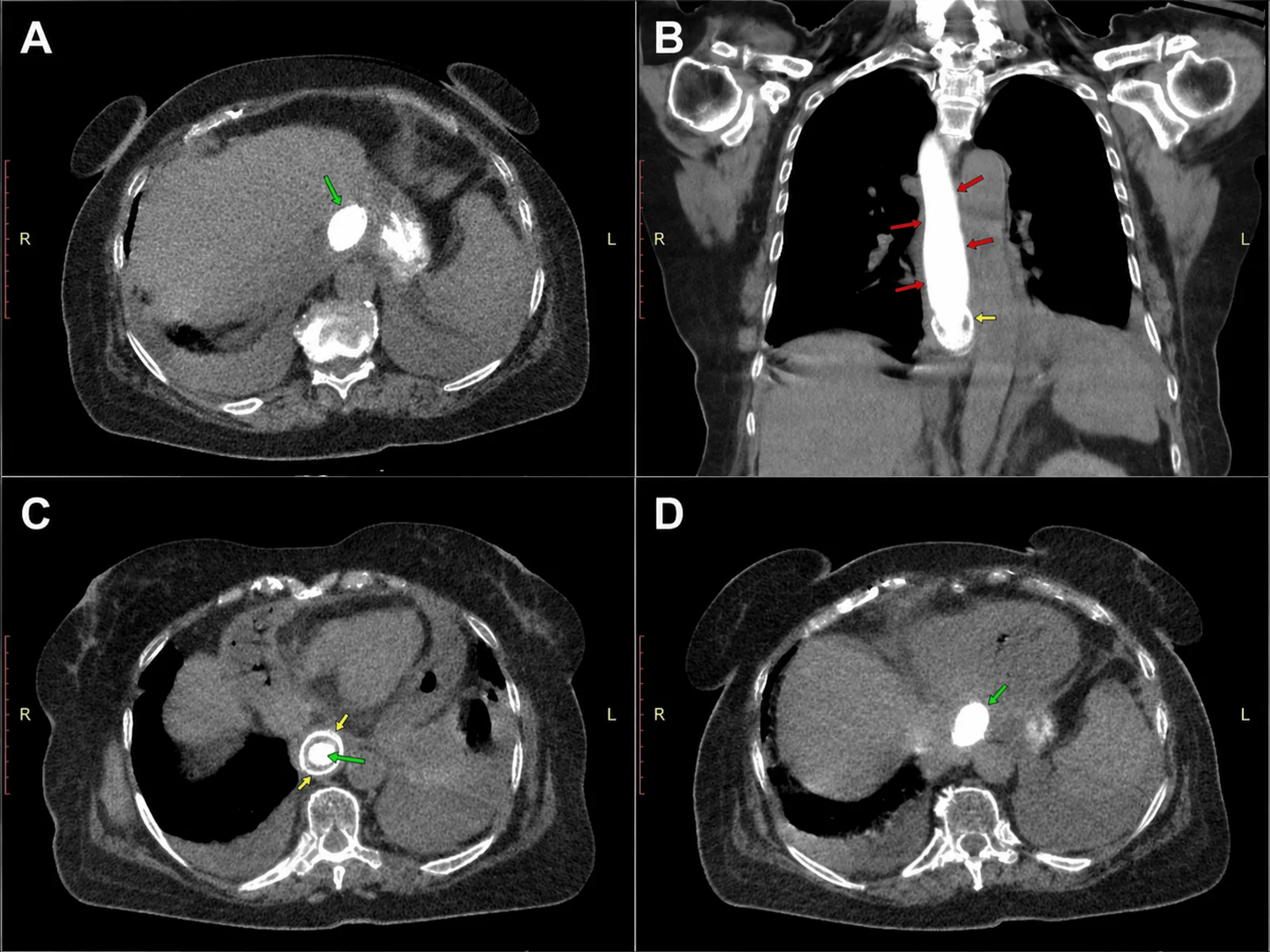
Contrast-enhanced chest computed tomography (CT) with oral contrast demonstrating opacification of the esophageal lumen without evidence of contrast extravasation or a visible fistulous communication. A: Axial CT image demonstrating oral contrast within the esophageal lumen (green arrow). B: Coronal CT image demonstrating oral contrast within the esophageal lumen (red arrows) and the esophageal stent (yellow arrow). C: Axial CT image demonstrating oral contrast within the esophageal lumen (green arrow) and the esophageal stent (yellow arrows). No contrast extravasation or fistulous communication is identified. D: Axial CT image demonstrating persistent oral contrast within the esophageal lumen (green arrow).

Given the absence of a demonstrable communication on CT, we hypothesized that an esophagopericardial fistula had formed during esophageal stent implantation two years earlier and had subsequently sealed spontaneously.

The patient was admitted to the hospital. An infectious disease consultation was obtained, further diagnostic investigations were planned, and guideline-directed heart failure therapy was initiated to relieve congestion.

Cardiac magnetic resonance imaging (CMR) was subsequently performed and demonstrated features consistent with constrictive physiology, including septal bounce. CMR revealed no imaging findings suggestive of infiltrative or storage disease or active pericardial inflammation; late gadolinium enhancement was not noted in the myocardium or pericardium (Video 2).

**Video 2:** Cardiac magnetic resonance imaging demonstrated septal bounce, consistent with constrictive physiology.

The patient subsequently underwent surgery via median sternotomy. The pericardial cavity was opened, revealing a large amount of firm, white, cheese-like material, which was evacuated. A pericardiectomy was performed, and the excised pericardial tissue together with the pericardial material was sent for microbiological and histopathological examination (Figure 7).

**Figure 7:**
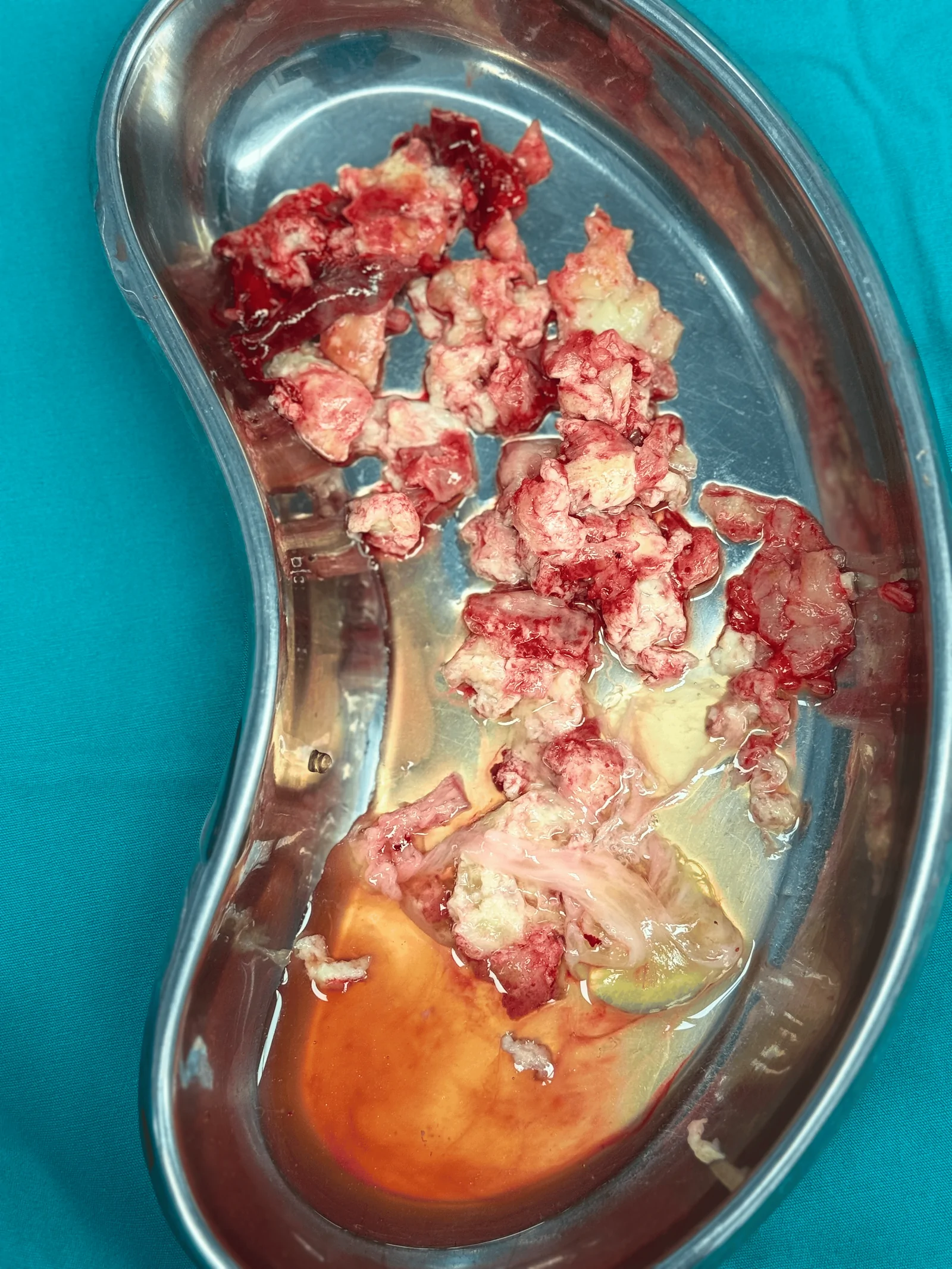
Material derived from pericardial cavity

Microbiological examination of the pericardial specimen isolated *Enterobacter cloacae* susceptible to meropenem and piperacillin/tazobactam (Table 2). Accordingly, treatment with piperacillin/tazobactam was continued based on the antimicrobial susceptibility results. 

**Table 2:** Microbiological examination and antimicrobial susceptibility results MIC, minimum inhibitory concentration.

Organism	Enterobacter cloacae	
Antibiotic	MIC	Susceptibility
Meropenem	<0.25	Susceptible
Piperacillin/tazobactam	<4	Susceptible
Cefepime	4	Intermediate
Ceftazidime	>32	Resistant
Ceftriaxone	>64	Resistant
Ciprofloxacin	0.5	Intermediate
Trimethoprim/sulfamethoxazole	<20	Susceptible
Ertapenem	<0.12	Susceptible
Amikacin	<1	Susceptible
Amoxicillin/clavulanate	>64	Resistant
Ampicillin	>32	Resistant
Gentamicin	<1	Susceptible

Histopatological examination of the resected pericardium demonstrated chronic inflammation, extensive fibrosis, and distrophic calcification. All these results were consistent with constrictive pericarditis. 

Following surgical intervention, the patient's CRP levels and leukocytosis progressively decreased. The trends in CRP and white blood cell count throughout the course of treatment are shown in Figure 8 and Table 3.

**Figure 8:**
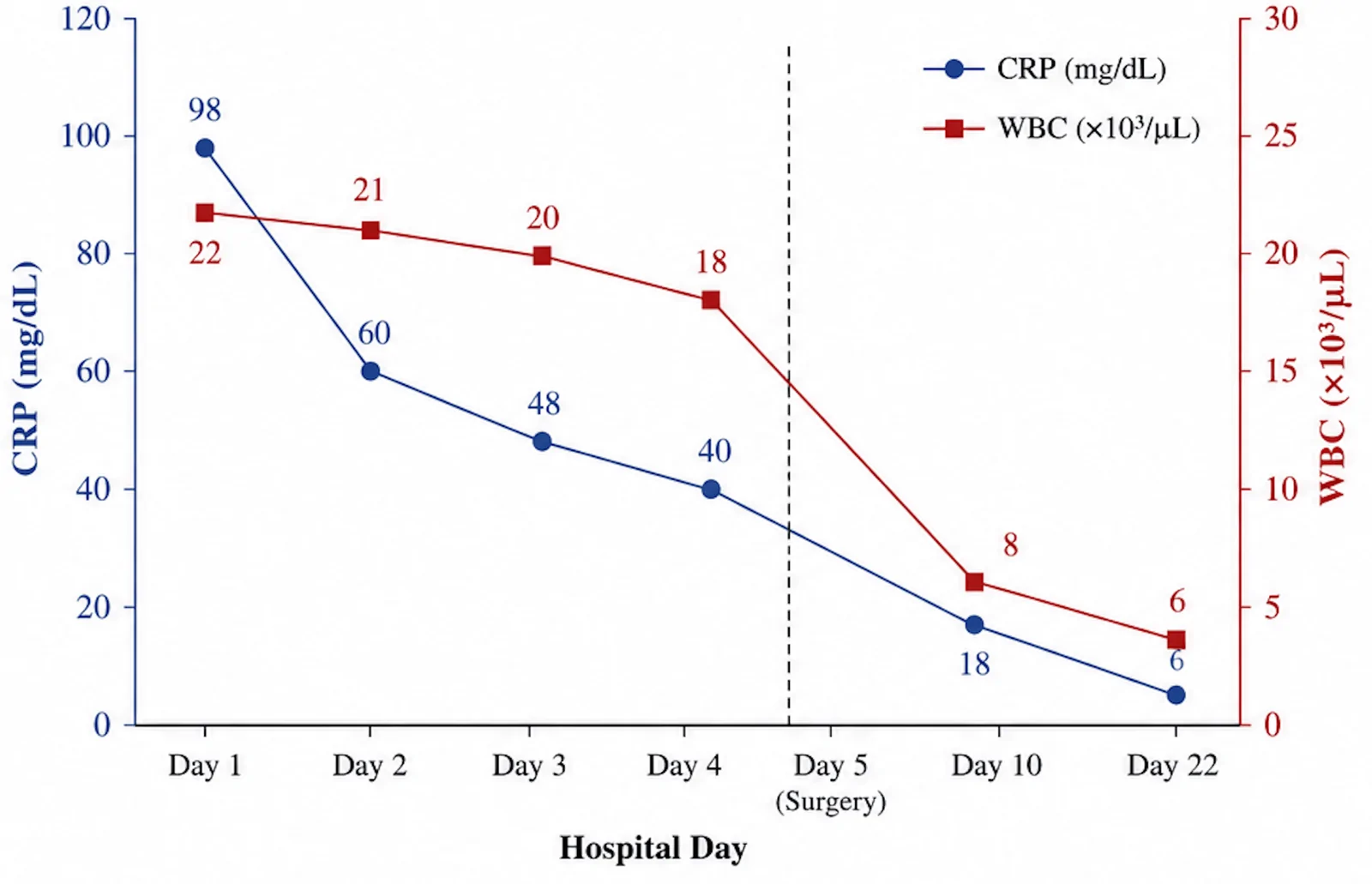
Trends of C-reactive protein (CRP) and white blood cells (WBCs) during hospital course

**Table 3:** Trends of C-reactive protein (CRP) and white blood cell (WBC) during hospital course

Hospital day	CRP (mg/dL)	WBC (×10³/µL)
Day 1	98	22
Day 2	60	21
Day 3	48	20
Day 4	40	18
Day 5	Surgery	Surgery
Day 10	18	8
Day 22	6	6

 In addition, the patient's signs and symptoms of heart failure resolved (Figure 9).

**Figure 9:**
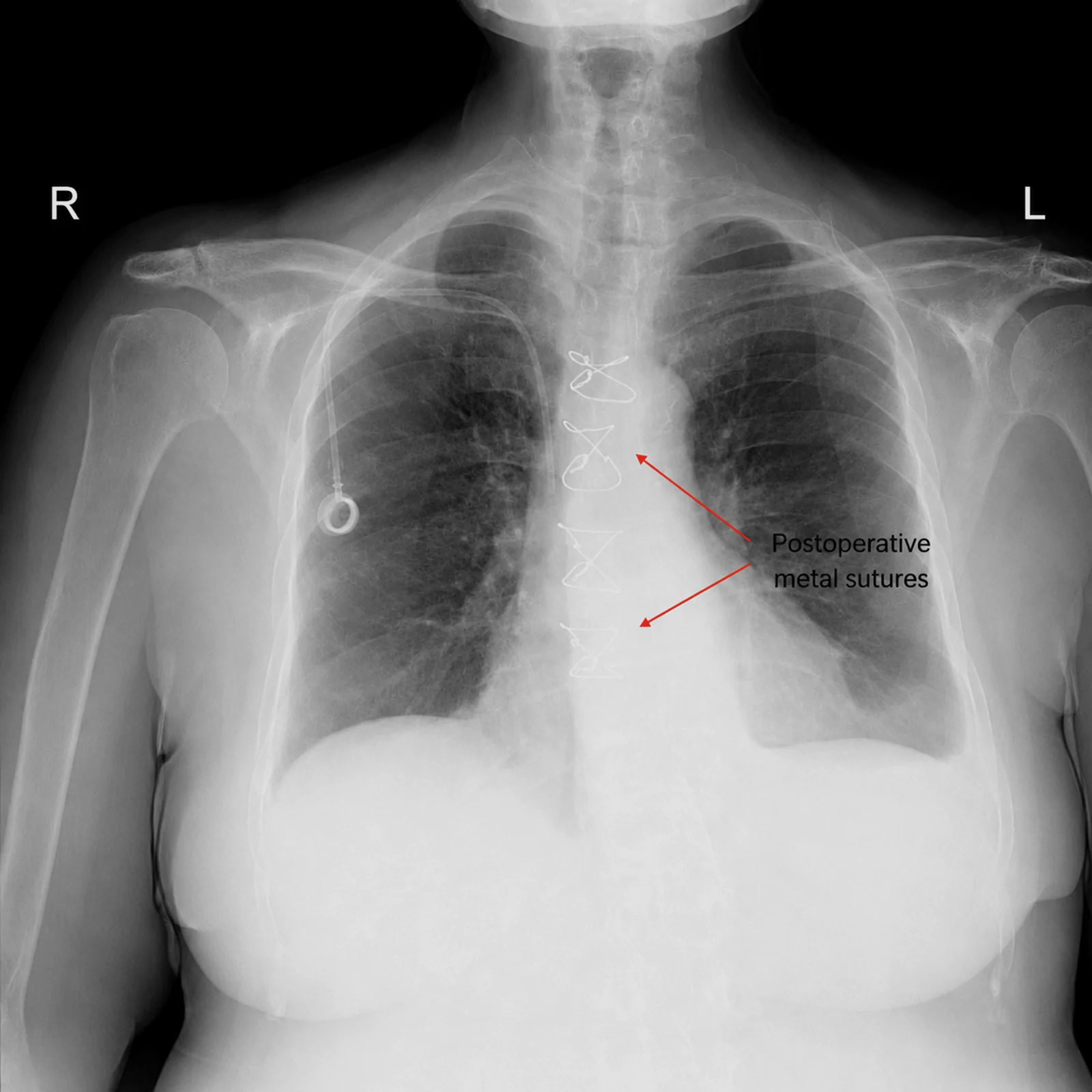
Postoperative chest radiography demonstrating almost complete resolution of the bilateral pleural effusions. Arrows show postoperative metal sutures.

Postoperative echocardiography revealed no significant pericardial effusion. No respiratory variation was observed in transmittal and transtricuspid Doppler inflow velocities (Figure 10).

**Figure 10:**
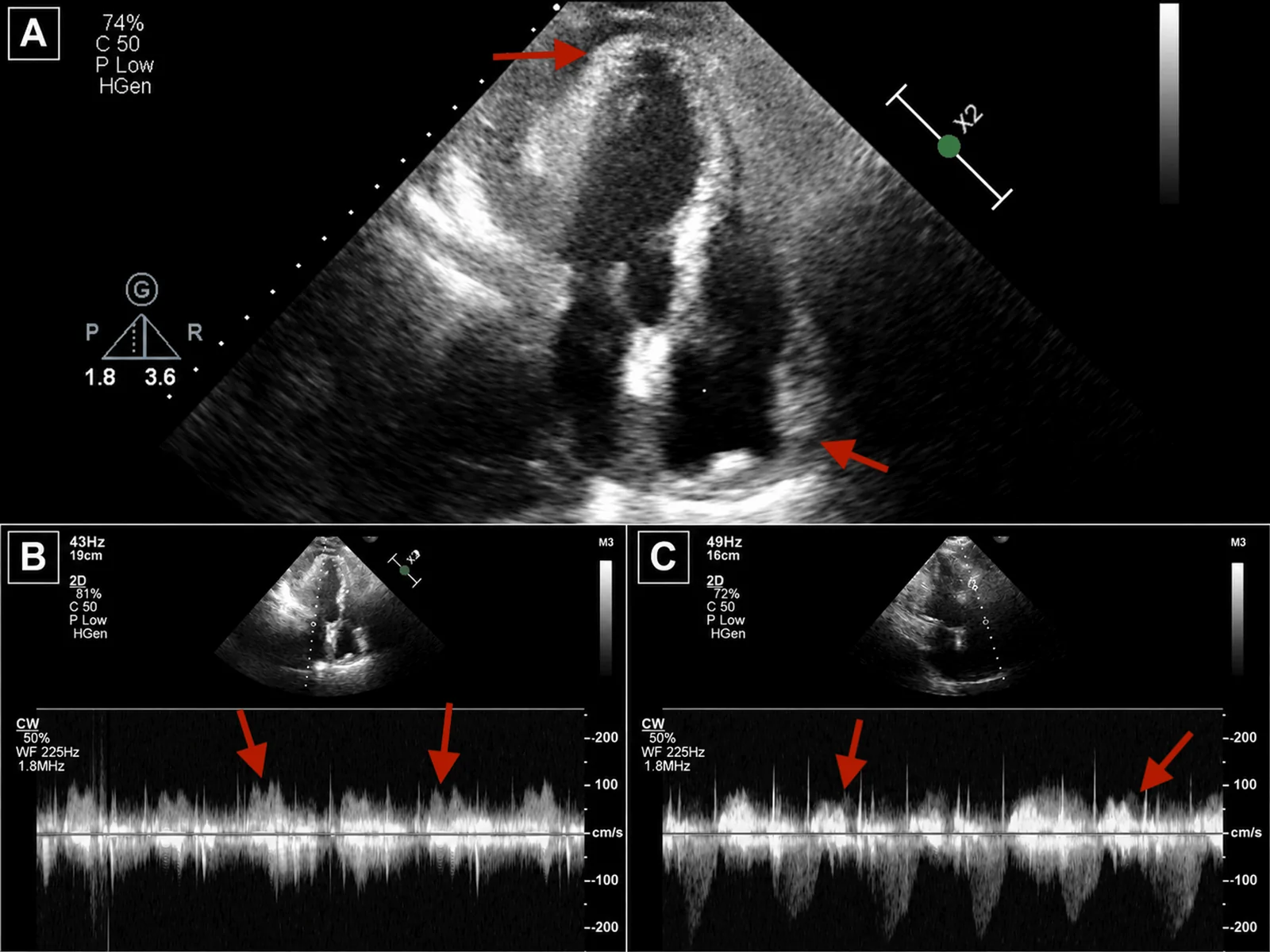
Postoperative transthoracic echocardiography findings A: Apical four-chamber view demonstrating no significant pericardial effusion (arrows). B: Continuous-wave Doppler of the transmitral inflow showing no significant respiratory variation (arrows). C: Continuous-wave Doppler of the transtricuspid inflow demonstrating no significant respiratory variation (arrows).

The patient is currently at the six-month follow-up and remains asymptomatic without any guideline-directed heart failure medicines.

## Discussion

Constrictive physiology and restrictive cardiomyopathy are two distinct pathological entities that often present with similar clinical manifestations, including progressive dyspnea, right-sided heart failure, peripheral edema, pleural effusions, and preserved left ventricular systolic function [1,7]. Differentiating between these conditions is essential because their management and prognosis differ substantially. While constrictive pericarditis may be potentially reversible with pericardiectomy, restrictive cardiomyopathy is primarily treated medically, and surgical intervention has no established role [1].

Both conditions are characterized by impaired ventricular filling and elevated diastolic pressures. However, constrictive physiology results from a rigid, non-compliant pericardium that limits ventricular expansion, whereas restrictive cardiomyopathy arises from intrinsic myocardial disease. This fundamental difference produces characteristic hemodynamic findings. In constrictive physiology, ventricular interdependence is exaggerated, leading to respiratory variation in ventricular filling, septal bounce, and dissociation between intrathoracic and intracardiac pressures. In contrast, restrictive cardiomyopathy demonstrates impaired myocardial relaxation without significant ventricular interdependence or marked respiratory variation in transvalvular flow velocities [7].

Echocardiography remains the first-line imaging modality for differentiating these entities. Characteristic findings supporting constrictive physiology include septal bounce, marked respiratory variation in transmitral inflow velocities, preserved or increased medial mitral annular early diastolic velocity (annulus paradoxus), and a dilated inferior vena cava with reduced inspiratory collapse [7,8]. In our patient, transthoracic echocardiography demonstrated a fibrinous pericardial effusion, septal bounce, respiratory variation of mitral inflow, and a dilated inferior vena cava, all supporting constrictive physiology rather than restrictive cardiomyopathy. CMR further confirmed septal bounce, strengthening the diagnosis [8].

An additional distinguishing feature in this case was the identification of pneumopericardium on CT. Air within the pericardial space is an uncommon finding and should prompt evaluation for prior cardiac intervention, trauma, infection, or communication with adjacent structures. Given the patient's history of esophageal stent implantation two years earlier, an esophagopericardial fistula was strongly suspected. Although oral and intravenous contrast-enhanced CT demonstrated no active communication between the esophagus and the pericardial cavity, the presence of pneumopericardium together with the clinical history suggested that a fistulous tract had likely developed following stent implantation and subsequently closed spontaneously. Chronic pericardial inflammation resulting from this previous communication most likely led to progressive pericardial fibrosis and the eventual development of constrictive physiology.

Although invasive hemodynamic assessment by right and left heart catheterization is considered the reference standard for confirming constrictive physiology, it was not performed in our patient. The diagnosis was established based on concordant findings from multimodality imaging, including transthoracic echocardiography, CMR, and CT. These studies consistently demonstrated features of constrictive physiology and provided an anatomical explanation for the underlying etiology. As the imaging findings were considered diagnostic and invasive hemodynamic assessment was not expected to alter clinical decision-making, cardiac catheterization was deferred [9].

Esophagopericardial fistula is an exceptionally rare but potentially fatal complication of esophageal disease and interventions. Most reported cases present acutely with purulent pericarditis, cardiac tamponade, or sepsis, requiring urgent surgical management [10]. Delayed presentation as chronic constrictive physiology has rarely been described. Therefore, clinicians should maintain a high index of suspicion when pneumopericardium is identified in patients with a history of esophageal instrumentation, even when active contrast leakage is absent [11].

An additional noteworthy finding in our case was the isolation of *Enterobacter cloacae* from the pericardial specimen. *Enterobacter cloacae* is a gram-negative bacillus belonging to the normal gastrointestinal microbiota and is an uncommon cause of bacterial pericarditis [12-14]. In the absence of recent cardiac surgery or penetrating trauma, its isolation strongly suggests contamination of the pericardial space by gastrointestinal flora [14]. Given the patient's history of esophageal stent implantation, the microbiological findings further support the hypothesis that a transient esophagopericardial fistula had developed, allowing translocation of enteric microorganisms into the pericardial cavity. Although no active communication was demonstrated on contrast-enhanced CT, spontaneous closure of the fistulous tract before presentation may explain the absence of contrast leakage despite persistent chronic pericardial inflammation.

Chronic low-grade infection and the resulting inflammatory response may have contributed to progressive pericardial fibrosis and calcification, ultimately leading to constrictive physiology. This proposed mechanism is supported by the intraoperative finding of dense fibrotic, cheese-like pericardial material together with the favorable clinical response following surgical pericardiectomy and targeted antimicrobial therapy.

## Conclusions

This case highlights an exceptionally rare delayed presentation of constrictive pericarditis with purulent pericardial infection following previous esophageal stent implantation. The presence of intrapericardial air strongly suggested a prior esophagopericardial communication despite the absence of an active fistulous tract on contrast-enhanced CT, raising the possibility of a spontaneously sealed fistula. Multimodality imaging, combined with surgical exploration, microbiological culture, and histopathological examination, was essential for establishing the diagnosis. Clinicians should maintain a high index of suspicion for late infectious pericardial complications in patients with a history of esophageal stenting, even years after the initial intervention, as timely surgical management and targeted antimicrobial therapy can result in favorable clinical outcomes.
